# In patients with severe asthma with eosinophilia in reslizumab clinical trials, high peripheral blood eosinophil levels are associated with low FEV_1_ reversibility

**DOI:** 10.1186/s13223-020-00424-2

**Published:** 2020-04-19

**Authors:** J. Christian Virchow, Lisa Hickey, Evelyn Du, Margaret Garin

**Affiliations:** 1 Departments of Pneumology/Intensive Care Medicine, University Medical Center, University of Rostock, Rostock, Germany; 2 Former Employee of Teva Branded Pharmaceutical Products R&D Inc., West Chester, PA USA; 3 Teva Branded Pharmaceutical Products R&D Inc., West Chester, PA USA

**Keywords:** Asthma, Clinical trials, Eosinophils, Lung function

## Abstract

**Background:**

A post hoc analysis of two randomized, placebo–controlled, Phase 3 trials of intravenous reslizumab, an anti-interleukin-5 (IL-5) biologic for severe eosinophilic asthma.

**Methods:**

Relationships between baseline blood eosinophil levels (EOS), forced expiratory volume in 1 s (FEV_1_) reversibility to β_2_-agonists and treatment outcomes were assessed.

**Results:**

Mean baseline FEV_1_ reversibility was numerically lower among patients with high (≥ 400 cells/µL) versus low baseline EOS. Reslizumab produced clinically significant improvement in FEV_1_, exacerbation rates and patient-reported outcomes after 52 weeks, including in patients with high EOS and low FEV_1_ reversibility (≤ 14%) to β_2_-agonists at baseline.

**Conclusions:**

Clinical trial eligibility criteria stipulating minimum FEV_1_ reversibility to β_2_-agonists of ≥ 12% might exclude patients who would benefit from treatment with anti-IL-5 biologics.

## Introduction

Severe non-allergic, adult-onset or intrinsic asthma is frequently associated with pronounced eosinophilia [1–4]. Interleukin-5 (IL-5) is a potent activator of eosinophils and enhances their viability [5]. IL-5 activity has been demonstrated to inversely correlate with pulmonary function in patients with asthma [4], and anti-IL-5 treatment has been shown to improve asthma control in patients with severe asthma and eosinophilia [6].

Reslizumab is an IgG4-kappa humanized monoclonal antibody targeting IL-5 [7]. In Phase 3 clinical trials intravenous (IV) reslizumab dosed at 3 mg/kg once every 4 weeks (q4w) was associated with a significant reduction in the risk of clinical asthma exacerbations (CAEs) and improved asthma control, lung function, and quality of life in patients with inadequately controlled asthma with blood eosinophil levels (EOS) ≥ 400 cells/µL and a history of CAEs [8]. Reslizumab has been indicated as add-on maintenance treatment for adult patients with severe eosinophilic asthma [9].

Among the entry criteria for these trials, patients had to have EOS ≥ 400 cells/µL at screening. In addition, based on traditional concepts to substantiate the presumed diagnosis of asthma, all patients had to demonstrate a reversibility of their forced expiratory volume in 1 s (FEV_1_) in response to the inhalation of a β_2_-agonist (albuterol 200 µg) of ≥ 12%. A recent longitudinal cohort study in young adults, with and without asthma, has shown that elevated EOS are associated with airflow obstruction [10].

We therefore hypothesized that any substantial increase in FEV_1_ following the administration of a β_2_–agonist might reflect asthma dominated by smooth muscle contraction rather than eosinophilic inflammation. On the other hand, a poor response to β_2_–agonists in patients with severe asthma and eosinophilia might predict a better response to anti–IL-5 therapy. To substantiate this we performed a post hoc analysis of data from two Phase 3 trials of IV reslizumab (NCT01287039 and NCT01285323) [8] to assess the relationship between EOS, reversibility of airway obstruction and treatment response to reslizumab therapy in a well-characterized population of patients with inadequately controlled moderate–to–severe asthma with EOS ≥ 400 cells/µL.

## Methods

### Study design and patients

The two duplicate trials enrolled patients aged 12–75 years with inadequately controlled asthma (Asthma Control Questionnaire-7 [ACQ-7] score ≥ 1.5) on medium-to-high doses of inhaled corticosteroids (ICS), and who had screening EOS ≥ 400 cells/μL, ≥ 1 CAE in the previous year, and FEV_1_ reversibility of ≥ 12% with albuterol [8]. The selection criteria for FEV_1_ reversibility were chosen based on National Asthma Education and Prevention Program guidelines available at the time of study design [11].

Both trials were conducted in accordance with Good Clinical Practice guidelines, the Declaration of Helsinki, and local regulatory requirements. All patients provided written informed consent, and the relevant health authorities and local ethics committees or institutional review boards approved the study protocols.

Following a 2–4-week screening period, patients were randomized (1:1) to receive IV reslizumab (3.0 mg/kg) or matching placebo q4w for 52 weeks. Patients continued their usual asthma treatment during the screening, run-in and treatment periods. Pre-bronchodilator spirometry, Asthma Symptom Utility Index (ASUI), and ACQ-7 were assessed q4w at the scheduled clinic visits, from day of randomization to the end of treatment. Possible cases of CAEs were assessed by questioning of the patient at every scheduled monthly visit. Asthma Quality of Life Questionnaire (AQLQ) score was assessed at baseline and weeks 16, 32, and 52.

### Outcome measures

FEV_1_ reversibility at baseline (during screening) was assessed according to EOS category at baseline (day of first dose). Categories for baseline FEV_1_ reversibility were arbitrarily set at < 14%, 14 to < 16%, 16 to < 20% and ≥ 20%, with baseline EOS categories set arbitrarily at < 150 cells/µL, 150 to < 400 cells/µL, 400 to < 700 cells/µL and ≥ 700 cells/µL. Given that blood eosinophil counts are known to be variable over time, assessment of blood eosinophil count at baseline allowed for selection of patients with persistently elevated blood eosinophils ≥ 400 cells/µL at two timepoints (screening and baseline).

The effect of reslizumab versus placebo on asthma clinical outcomes was also assessed in the subgroup with baseline low FEV_1_ reversibility (12–14%) and pooled high EOS (≥ 400 cells/µL). Assessment of lung function comprised FEV_1_, FEV_1_% predicted, forced vital capacity (FVC) and forced expiratory flow at 25–75% of pulmonary volume (FEF_25–75%_), and other asthma clinical outcomes assessed were CAEs, ACQ-7, AQLQ and ASUI.

### Statistical analysis

An analysis of covariance was used to model change from baseline at Week 52 in lung function and patient-reported outcomes (ACQ-7, AQLQ and ASUI) with fixed factors for treatment arm, sex, oral corticosteroid use at baseline (Yes or No), region (USA or Other), and a continuous covariate for height.

CAEs counted are those which occurred between the completion of the first dose of study drug and 2 weeks after the end of treatment/early withdrawal visit. CAE rates, CAE rate ratio, and confidence intervals (CIs) and *p* values are based on a negative binomial regression model adjusted for baseline usage of oral corticosteroid (Yes or No) and region (USA or other).

All analyses were conducted using SAS version 9.4 (SAS Institute Inc., Cary, NC, USA).

## Results

A total of 953 patients were randomized in the two duplicate Phase 3 studies (reslizumab: n = 477; placebo: n = 476). Patient demographics and clinical characteristics at baseline were similar between reslizumab and placebo groups (Table 1).

**Table 1:** Patient characteristics during the baseline period

Characteristic baseline subgroup	Placebo (N = 476)	Reslizumab (N = 477)
Mean age (SD), years	47.1 (14.3)	46.5 (13.8)
Females, n (%)	311 (65)	286 (60)
Mean body mass index (SD), kg/m^2^	27.5 (5.7)	27.4 (5.8)
Oral corticosteroid use, n (%)	73 (15)	73 (15)
LABA use, n (%)	383 (80)	397 (83)
High-dose ICS^a^ use, n (%)	208 (44)	203 (43)
Mean FEV_1_ (SD), mL	1965 (734)	2008 (763)
EOS < 150 cells/µL	1947 (643)	2317 (834)
EOS 150 to < 400 cells/µL	2101 (759)	2032 (734)
EOS 400 to < 700 cells/µL	1997 (795)	2091 (785)
EOS ≥ 700 cells/µL	1860 (655)	1846 (710)
FEV_1_ reversibility < 14%	2258 (735)	1975 (754)
FEV_1_ reversibility 14 to < 16%	2187 (666)	2180 (659)
FEV_1_ reversibility 16 to 20%	1944 (838)	2045 (821)
FEV_1_ reversibility ≥ 20%	1841 (678)	1979 (763)
Mean FEV_1_ predicted (SD), %	66.5 (19.4)	66.9 (20.0)
Mean FEV_1_ reversibility (SD), %	27.5 (21.1)	27.0 (15.8)
Mean FVC (SD), mL	3008 (1030)	3070 (1010)
Mean blood EOS (SD), cells/µL	655 (637)	654 (621)
Mean age of asthma onset (SD), years	27.8 (17.9)	27.3 (18.4)

^a^High-dose ICS use was defined as when one of the following was true at enrolment: fluticasone > 500 µg/day, mometasone > 440 µg/day, budesonide > 800 µg/day, ciclesonide > 320 µg/day, beclomethasone > 400 µg/day or triamcinolone > 2000 µg/day

*EOS* eosinophil, FEV_1_ forced expiratory volume in 1 s, *FVC* forced vital capacity, *ICS* inhaled corticosteroid, *LABA* long-acting beta agonist, *SD* standard deviation

### Baseline eosinophil categories and FEV_1_ reversibility

During the screening period, all patients were required to have EOS ≥ 400 cells/µL. However, on the day of the first reslizumab dose, 65 patients had EOS < 150 cells/µL, 179 patients had EOS 150 to < 400 cells/µL, 365 patients had EOS 400 to < 700 cells/µL, and 344 patients had EOS ≥ 700 cells/µL. At baseline, 149 patients had an FEV_1_ reversibility of < 14% (between 12 and 14%), 104 had reversibility between 14% and < 16%, 172 had reversibility of 16–20%, and 528 had reversibility of ≥ 20%. Across EOS subgroups, baseline mean FEV_1_ was numerically lowest in the EOS ≥ 700 cells/µL subgroup for both reslizumab and placebo (Table 1). There was no clear relationship between baseline mean FEV_1_ and FEV_1_ reversibility subgroup (Table 1). Baseline mean FEV_1_ was generally comparable between reslizumab and placebo treatment arms within patient subgroups (Table 1).

### Baseline FEV_1_ reversibility according to eosinophil group

**Fig. 1:**
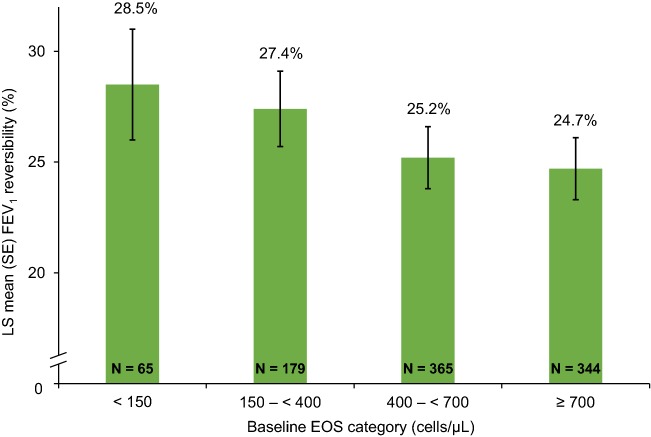
Mean baseline FEV_1_ reversibility according to baseline blood eosinophil category. *EOS* blood eosinophil level; *FEV*_*1*_ forced expiratory volume in 1 s; *LS* least-squares; *SE* standard error

**Fig. 2:**
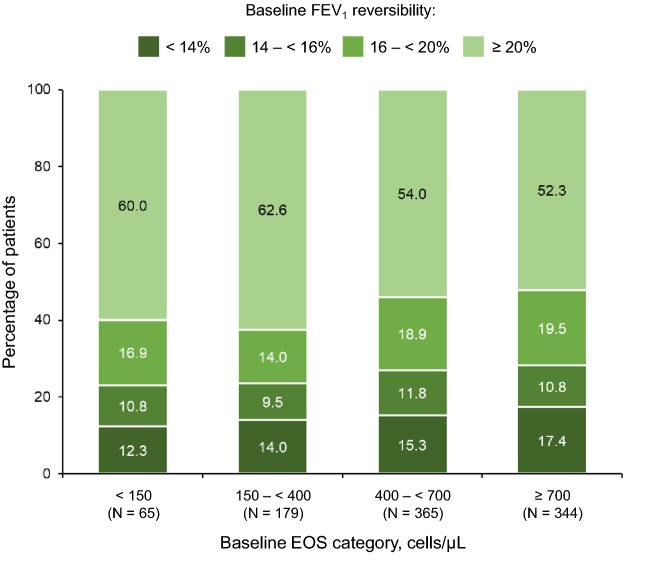
Proportions of patients in each category of baseline FEV_1_ reversibility according to baseline EOS. *EOS* blood eosinophil level; *FEV*_*1*_ forced expiratory volume in 1 s

Those patients who had low baseline EOS (< 150 cells/µL or 150 to < 400 cells/µL) had a higher mean FEV_1_ reversibility and a higher proportion of patients who were highly reversible to inhaled β_2_-agonists (≥ 20% reversibility, 60% and 62.6% of the subgroup populations) compared with patients with higher EOS (Fig. 1). The proportion of patients who responded relatively poorly to β_2_-agonists (< 14% improvement) was largest in the EOS ≥ 700 cells/µL group (17.4%) compared with other EOS groups, and this high EOS group had the numerically lowest mean reversibility (Fig. 2).

### Reslizumab treatment effect on lung function measures

**Fig. 3:**
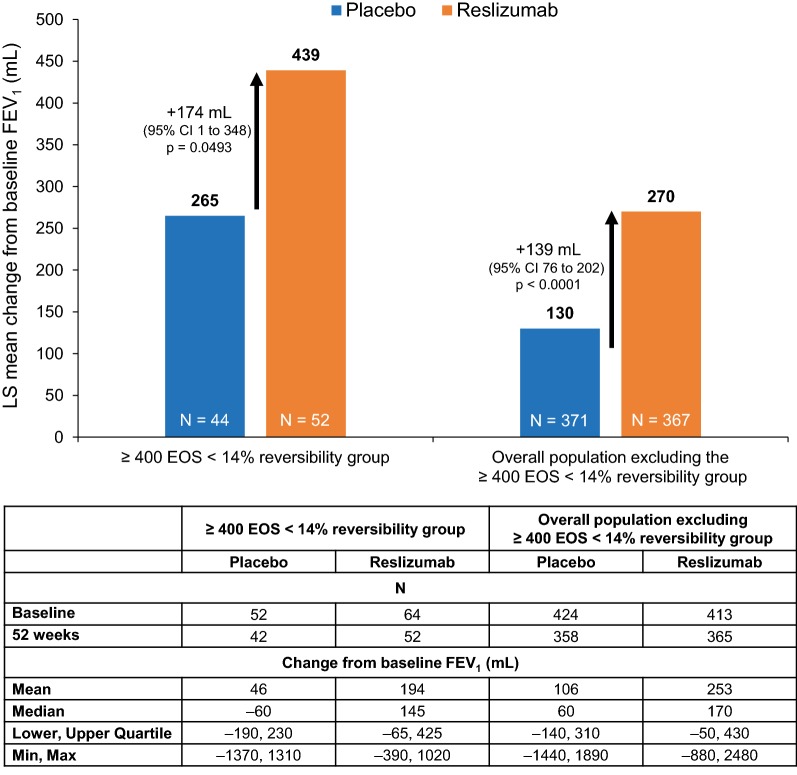
Change from baseline FEV_1_ at 52 weeks among patients with high EOS and low reversibility, compared with the overall population excluding these patients. *CI* confidence interval; *EOS* blood eosinophil level; *FEV*_*1*_ forced expiratory volume in 1 s; *IQR* interquartile range; *LS* least–squares

Figure 3 shows the observed treatment effects for reslizumab on FEV_1_ versus placebo at 52 weeks in the group comprising patients with high EOS and the lowest FEV_1_ reversibility (EOS ≥ 400 cells/µL, < 14% reversibility) compared with the remaining overall population excluding those with EOS ≥ 400 cells/µL and < 14% reversibility. Both groups experienced a clinically significant improvement in FEV_1_ at 52 weeks with reslizumab versus placebo (mean: +174 mL [95% CI 1–348] and +139 mL [95% CI 76–202], respectively). Interestingly, despite the relatively poor response to β_2_-agonists, in the EOS high/β_2_-agonist reversibility low group there was a marked improvement compared with placebo, with a numerically greater treatment effect compared with the remaining population. The absolute increase in FEV_1_ in mL from baseline after 52 weeks in the high EOS/low β_2_-agonist reversibility group was numerically higher than the change from baseline in the remaining patient population with both reslizumab treatment (mean: + 439 mL [standard error [SE] 105] and + 270 mL [SE 36]) and placebo (mean: + 265 mL [SE 98] and + 130 mL [SE 37]). However, numerical differences in treatment effect for FEV_1_ between the EOS high/β_2_ agonist reversibility low group and the remaining population did not reach statistical significance. Baseline values and treatment effects in these two groups on FEV_1_, FVC and FEF_25–75%_ are shown in Table 2.

**Table 2:** Change from baseline in lung function parameters after 52 weeks

	Placebo		Reslizumab		52-week treatment difference (reslizumab vs placebo) (95% CI)	*p* value
	Baseline	Change at 52 weeks	Baseline	Change at 52 weeks		
≥ 400 EOS < 14% reversibility group						
N	52	42	64	52		
FEV_1_, mL	2339 (107)	265 (98)	1945 (92)	439 (105)	174 (1 to 348)	0.0493
FEV_1_% predicted	73.3 (2.6)	6.7 (3.2)	67.4 (2.2)	13.8 (3.4)	7.1 (1.4 to 12.7)	0.0144
FVC, mL	3.5 (0.14)	271 (121)	2.9 (0.13)	419 (129)	148 (− 65 to 362)	0.1714
FEF_25–75%_, mL^a^	1.7 (0.14)	297 (133)	1.3 (0.090)	444 (141)	147 (− 88 to 383)	0.2172
Overall population excluding the ≥ 400 EOS < 14% reversibility group						
N	424	358	413	365		
FEV_1_, mL	1919 (35)	130 (37)	2018 (38)	270 (36)	139 (76 to 202)	< 0.0001
FEV_1_% predicted	65.7 (0.94)	4.7 (1.2)	66.8 (1.0)	9.1 (1.2)	4.4 (2.3 to 6.5)	< 0.0001
FVC, mL	2.9 (0.049)	157 (46)	3.1 (0.049)	288 (45)	131 (52 to 210)	0.0013
FEF_25–75%_, mL^b^	1.7 (0.29)	79 (53)	1.4 (0.043)	207 (51)	128 (39 to 217)	0.0047

Baseline data are mean (SE). All change from baseline data are LS mean (SE)

*CI* confidence interval, *EOS* blood eosinophil level, *FEF*_*25*–*75%*_ forced expiratory flow at 25–75% of pulmonary volume, *FEV*_*1*_ forced expiratory volume in 1 s, *FVC* forced vital capacity, *LS* least square, *SE* standard error

^a^FEF_25–75%_ data unavailable for n = 1 (placebo), n = 1 (reslizumab) patients

^b^FEF_25–75%_ data unavailable for n = 3 (placebo), n = 5 (reslizumab) patients

### Reslizumab treatment effect on other asthma clinical measures

At 52 weeks, mean annualized exacerbation rate was lower with reslizumab versus placebo in the high EOS/low β_2_-agonist reversibility group (0.63 vs 1.06, respectively; rate ratio 0.60 [95% CI 0.33, 1.09]; *p* = 0.0937) and in the remaining overall population excluding those with EOS ≥ 400 cells/µL and < 14% reversibility (0.59 vs 1.19, respectively; rate ratio 0.45 [95% CI 0.35, 0.57]; *p* < 0.0001) (Table 3). The CAE rate ratio for reslizumab versus placebo was numerically lower in the remaining overall population excluding those with EOS ≥ 400 cells/µL and < 14% reversibility compared to the high EOS/low β_2_-agonist reversibility group, although numerical differences did not reach statistical significance between groups.

**Table 3:** Clinical asthma endpoints

	Placebo		Reslizumab			
	Baseline	52 weeks	Baseline	52 weeks	Treatment effect	*p* value
≥ 400 EOS < 14% reversibility group						
					RR (reslizumab vs placebo) (95% CI)	
≥ 1 exacerbation, n (%)	NA	28 (53.8%)	NA	26 (40.6%)		
Adjusted exacerbation rate (over 52 weeks)^a^ (95% CI)	NA	1.52 (0.84, 2.72)	NA	0.91 (0.48, 1.72)	0.600 (0.330, 1.090)	0.0937

		Change from baseline at Week 52		Change from baseline at Week 52	52-week treatment difference (reslizumab vs placebo) (95% CI)	
N	52	44	64	52		
ACQ-7 score^b^	2.511 (0.117)	− 0.781 (0.158)	2.464 (0.111)	− 1.189 (0.159)	− 0.408 (− 0.753, − 0.064)	0.0206
AQLQ score^b^	4.259 (0.153)	0.872 (0.198)^c^	4.518 (0.140)	1.425 (0.200)	0.554 (0.148, 0.959)	0.0079
ASUI score^b^	0.634 (0.028)	0.118 (0.029)^c^	0.690 (0.024)	0.193 (0.029)^c^	0.075 (0.013, 0.137)	0.0174

Overall population excluding the ≥ 400 EOS < 14% reversibility group						
					RR (reslizumab vs placebo) (95% CI)	
≥ 1 exacerbation, n (%)	NA	209 (49.3%)	NA	125 (30.3%)		
Adjusted exacerbation rate over 52 weeks^a^ (95% CI)	NA	1.80 (1.37, 2.36)	NA	0.80 (0.61, 1.05)	0.446 (0.346, 0.575)	< 0.0001

		Change from baseline at Week 52		Change from baseline at Week 52	52-week treatment difference (reslizumab vs placebo) (95% CI)	
N	424	371	413	367		
ACQ-7 score^b^	2.708 (0.041)	− 0.805 (0.065)	2.637 (0.043)	− 1.141 (0.064)	− 0.336 (− 0.470, − 0.201)	< 0.0001
AQLQ score^b^	4.182 (0.053)	0.874 (0.077)^d^	4.297 (0.053)	1.171 (0.076)^c^	0.297 (0.150, 0.445)	< 0.0001
ASUI score^b^	0.630 (0.010)	0.145 (0.012)^e^	0.641 (0.010)	0.198 (0.012)^f^	0.053 (0.028, 0.078)	< 0.0001

*ACQ* Asthma Control Questionnaire, *AQLQ* Asthma Quality of Life Questionnaire, *ASUI* Asthma Symptom Utility Index, *CI* confidence interval, *EOS* blood eosinophil level, *LS* least square, *NA* not applicable, *SE* standard error

^a^Adjusted exacerbation rates and confidence intervals based on Negative Binomial regression model adjusted for baseline use of oral corticosteroids (yes or no) and geographical region (US or other)

^b^Baseline data are mean (SE). All change from baseline data are LS mean (SE)

^c^Data unavailable for one patient

^d^Data unavailable for three patients

^e^Data unavailable for five patients

^f^Data unavailable for four patients

Baseline values and treatment effects on ACQ, AQLQ and ASUI in the high EOS/low β_2_-agonist reversibility group and the remaining overall population excluding those with EOS ≥ 400 cells/µL and < 14% reversibility are shown in Table 3. Similar to the lung function findings, we observed a numerically greater treatment effect for reslizumab versus placebo across the asthma clinical outcomes in the high EOS/low β_2_-agonist reversibility group than in the remaining overall population excluding those with EOS ≥ 400 cells/µL and < 14% reversibility, with no statistically significant differences between groups.

## Discussion

Eosinophilia is an important but often variable feature of bronchial asthma, which is highly responsive to corticosteroids and anti-IL-5 therapy. Based on our observation we can postulate that patients with the highest EOS were more likely to have a relatively poor reversibility to β_2_-agonists, but were as likely to have a marked response to treatment with the anti-IL-5 antagonist reslizumab as the remaining population.

Unfortunately, due to standard asthma inclusion criteria for reversibility used in this study, no patients with EOS ≥ 400 cells/µL *and* a β_2_-agonist reversibility of ≤ 12% were studied. Based on our observed results it can be speculated, however, that these patients might have benefited from treatment with reslizumab as the lack of FEV_1_ increase following the administration of a β_2_-agonist might reflect asthma dominated by eosinophilic inflammation. Therefore, this population should be specifically investigated in a future study. At present, our data suggest that high β_2_-agonist reversibility might not be the best predictor of response to anti-IL-5 treatment while low β_2_-reversibility (in the presence of a marked eosinophilia) might be indicative of eosinophil-dependent asthma. Future studies should therefore test if the combination of the two ‘biomarkers’, namely a low β_2_-agonist reversibility together with a high peripheral blood eosinophilia, might be a better predictor of response to anti-IL-5 therapy than either parameter alone or other markers such as fractioned exhaled nitric oxide currently used to predict treatment responses in asthma. Finally, our observation suggests that in asthma low β_2_-agonist reversibility in the presence of eosinophilia should not lead to the assumption of an irreversibility of airflow obstruction, but in contrast might be indicative of persistent eosinophilic inflammation rather than ‘remodeling’, which might predict a marked responsiveness to anti-inflammatory treatment with corticosteroids and/or anti-IL-5 treatments.

In this study, we noted a large increase in FEV_1_ from baseline in the placebo groups, particularly in the EOS high/β_2_-agonist reversibility low group. The marked improvement in FEV_1_ after 52 weeks in the EOS high/β_2_-agonist reversibility low group receiving placebo may be due to a ‘trial effect’ including increased adherence to ICS, which might preferentially result in improvement in eosinophilic patients. However, the improvement on reslizumab was numerically better in this group than in the remaining patients, suggesting that a possibly good response in FEV_1_ to ICS does not seem to interfere with the actions of reslizumab on airflow obstruction in eosinophilic patients.

### Limitations of the study

Our study is limited by the post hoc nature of our analysis and the relatively small number of patients in the subgroup of interest (n = 52 in the group with the lowest reversibility to β_2_-agonists treated with reslizumab). Furthermore, our hypothesis that the biomarker combination of high peripheral blood eosinophilia/low β_2_-agonist reversibility might be an ideal predictor of response to reslizumab is limited by the inclusion criteria of FEV_1_ reversibility of ≥ 12% precluding assessment of patients with lower baseline reversibility values.

## Conclusion

Higher baseline EOS are associated with numerically lower FEV_1_ reversibility in patients with inadequately controlled asthma with eosinophilia. Reslizumab treatment resulted in clinically significant improvements in asthma clinical outcomes in patients with high EOS and low FEV_1_ reversibility at baseline. Therefore, reslizumab may preferentially reverse airway obstruction which appears ‘fixed’ to β_2_-agonists (albuterol) due to action on IL-5 and airway eosinophils. The exclusion of patients with airflow obstruction poorly responsive to β_2_-agonists from clinical trials of biologics for severe asthma may result in an under-representation of patients with enhanced inflammation with eosinophils who might in fact particularly benefit from this treatment.

## Data Availability

Additional data available on request.

## Abbreviations

ACQ-7: Asthma Control Questionnaire
AQLQ: Asthma Quality of Life Questionnaire
ASUI: Asthma Symptom Utility Index
CAE: Clinical asthma exacerbation
CI: Confidence interval
EOS: Blood eosinophil level
FEF_25–75%_: Forced expiratory flow at 25–75% of pulmonary volume
FEV_1_: Forced expiratory volume in 1 s
FVC: Forced vital capacity
ICS: Inhaled corticosteroid
IL-5: Interleukin-5
IV: Intravenous
LS: Least square
q4w: Every 4 weeks
SE: Standard error
